# Clinical Low Vision Rehabilitation Interventions in Age-Related Macular Degeneration: A Case Report

**DOI:** 10.7759/cureus.109419

**Published:** 2026-05-22

**Authors:** Nabila Y Al-Tamimi, Mohammed Amer, Fadila Nour Elrihen Graini

**Affiliations:** 1 Department of Ophthalmology, Hamad Medical Corporation, Doha, QAT; 2 College of Medicine, Qatar University, Doha, QAT

**Keywords:** age-related macular degeneration (amd), dry age-related macular degeneration, galilean telescope, low vision rehabilitation, optical aids, optometric management, wet age related macular degeneration

## Abstract

A 70-year-old female with bilateral age-related macular degeneration (AMD) - wet AMD in the right eye and dry AMD in the left eye - presented with reduced unaided distance visual acuity of 20/200 (6/60) and near visual acuity of 3.2M (20/160, 6/48) OU. Retinoscopy and subjective refraction showed a prescription of -2.25 -2.50 × 175 in the right eye and +0.50 -1.50 × 120 in the left eye, achieving corrected visual acuities of 20/125 (6/37.5) OU. With an additional +3.00 diopters sphere for near tasks, the patient was able to read up to 2.5M (20/125, 6/37.5), indicating a coexisting refractive error. Amsler grid testing revealed central metamorphopsia and scotomas OU. Optical coherence tomography (OCT) confirmed bilateral drusen, retinal pigment epithelium (RPE) atrophy, and vitreomacular adhesion (VMA), with findings consistent with AMD. Functional contrast sensitivity was reduced (20/100, 6/30) OU, and visual field testing showed central scotomas and metamorphopsia.

Low vision rehabilitation included the prescription of a 2.5× binocular Galilean telescope (Eschenbach 1639; Eschenbach Optik GmbH, Nuremberg, Germany) for distance, achieving 20/40 (6/12) vision OU, and a 5× illuminated hand magnifier (Eschenbach Mobilux 15105; Eschenbach Optik GmbH) for near tasks, enabling reading of 1.0M print (approximately 20/50, equivalent to 6/18+). Environmental modifications and contrast-enhancing filters were recommended. Nutritional counseling and ongoing monitoring were initiated, and the patient was educated on self-assessment with the Amsler grid. At three-month follow-up, visual function remained stable, and low vision strategies continued to support functional independence.

This case underscores the importance of early low vision intervention, multidisciplinary care, and personalized rehabilitation strategies in maintaining quality of life in patients with AMD.

## Introduction

Age-related macular degeneration (AMD) is a leading cause of vision loss among older adults, particularly in developed countries. It primarily affects the macula, the central region of the retina responsible for sharp, central vision. The dry form of AMD - also referred to as non-neovascular or atrophic AMD - is the more common type, accounting for approximately 80%-90% of cases [1]. In contrast, the wet form of AMD is more severe and is characterized by choroidal neovascularization, which may cause rapid central vision loss if left untreated [1]. Unlike the rapid vision loss seen in the wet form, dry AMD progresses gradually and may remain asymptomatic in its early stages. It is characterized by the slow degeneration of macular cells and the accumulation of drusen, small yellow deposits located beneath the retina [2]. Over time, this process can lead to a progressive decline in central vision, making everyday tasks such as reading, driving, and facial recognition increasingly difficult.

Low vision rehabilitation plays an important role in improving functional independence in patients with advanced AMD by using optical aids and adaptive strategies.

This case report describes a patient with right eye neovascular (wet) AMD and left eye non-neovascular (dry) AMD, highlighting clinical findings, diagnostic evaluation, and the role of individualized low vision rehabilitation in improving functional vision and daily activities.

## Case presentation

Initial visit (November 07, 2023)

A 70-year-old female presented with a chief complaint of constant blurry vision affecting both near and distance vision, accompanied by glare. She reported difficulty performing daily activities such as reading books and watching television, expressing a strong desire to improve her vision and regain independence at home. Her unaided distance visual acuity using the Snellen Visual Acuity Chart was 20/200 (6/60) in both eyes, and unaided near visual acuity using the Lea Number Visual Near Acuity Chart was 3.2M, equivalent to 20/160 (6/48) binocularly. Retinoscopy and subjective refraction revealed a prescription of -2.25 -2.50 × 175 in the right eye and +0.50 -1.50 × 120 in the left eye, resulting in corrected visual acuities of 20/125 (6/37.5) in both eyes. With an added +3.00 diopter sphere for near tasks, the patient was able to read up to 2.5M (20/125, 6/37.5).

Amsler grid testing revealed distortions, gaps, and wavy lines in the grid pattern in both eyes, indicative of macular pathology. Contrast sensitivity testing using the CSV-1000 Contrast Sensitivity Test (VectorVision, Greenville, OH, USA) demonstrated reduced function in both eyes, with functional acuity recorded at 20/100 (6/30). Visual field testing using the ZEISS Humphrey Field Analyzer (30-2) (Carl Zeiss Meditec, Jena, Germany) showed characteristic findings of AMD, including central scotomas, decreased sensitivity to light stimuli, and variably distributed scotomas in monocular and binocular fields. 

The anterior segment examination was unremarkable apart from mild bilateral cataracts. There was no history of ocular surgery, systemic illness, allergies, smoking, or recreational drug use. The patient was alert, oriented to person, place, and time, and her mood was appropriate. Family history was negative for diabetes mellitus, hypertension, cardiac disease, or other chronic illnesses.

Based on the clinical presentation and diagnostic testing, a diagnosis of AMD was established. The patient was counseled regarding her condition and the potential benefits of low-vision aids. A plan was discussed to prescribe a 2.5× binocular Galilean telescope for distance magnification (Eschenbach 1639; Eschenbach Optik GmbH) and a 5× (20-diopter) illuminated handheld magnifier for near tasks (Eschenbach Mobilux 15105; Eschenbach Optik GmbH), with the goal of improving functional vision and quality of life.

Slit lamp biomicroscopy

**Figure 1:**
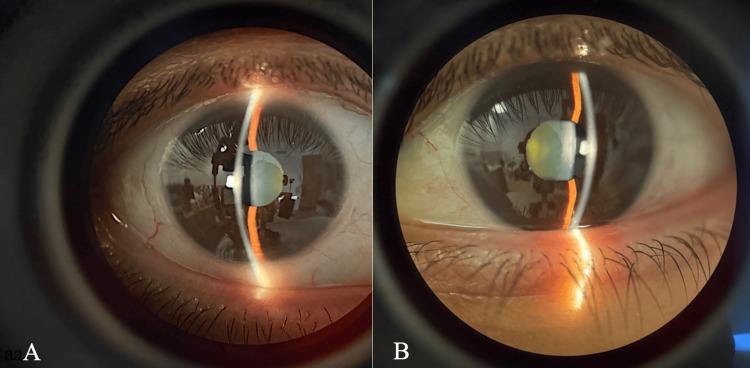
(A, B) Slit Lamp Biomicroscopy Findings (A) Right eye (OD); (B) Left eye (OS). Examination showed normal adnexa, lashes, and conjunctivae bilaterally. Both eyes exhibited clear irides, Grade 1 nuclear sclerosis, and Grade 0 conjunctival injection. The limbus was clear (Grade 0), and the tear prism height measured 0.2 mm in both eyes. Examination was performed using a Haag-Streit slit lamp biomicroscope (Haag-Streit AG, Köniz, Switzerland).

Examination revealed normal adnexa, lashes, puncta, and palpebral and bulbar conjunctivae bilaterally. The eyelids were clean and free of debris in both eyes. Both eyes exhibited Grade 1 nuclear sclerosis. The irides were clear, and the bulbar conjunctivae showed Grade 0 injection bilaterally. The limbus appeared clear, graded as 0, and the tear prism height measured 0.2 mm in both eyes. Examination findings are shown in Figure 1. 

Fundus examination

Fundus assessment was performed using OPTOS Ultra-Wide Field (UWF) imaging (Optos plc, Nikon Corporation, Scotland, United Kingdom). In both eyes, the optic nerve heads demonstrated a cup-to-disc (C/D) ratio of 0.4, with well-defined margins, normal shape, and orange coloration. The neuroretinal rim (NRR) followed the ISNT rule (inferior > superior > nasal > temporal) in each eye. The arteriovenous (AV) ratio was approximately 2:3, with arteries appearing narrower than veins within normal physiological limits. The retinal vasculature appeared unremarkable.

The maculae of both eyes exhibited drusen. Additional drusen were observed throughout the peripheral fundus in all gaze positions. The fundus background showed a bilateral tigroid appearance, indicative of increased choroidal vessel visibility secondary to thinning of the retinal pigment epithelium (RPE).

**Figure 2:**
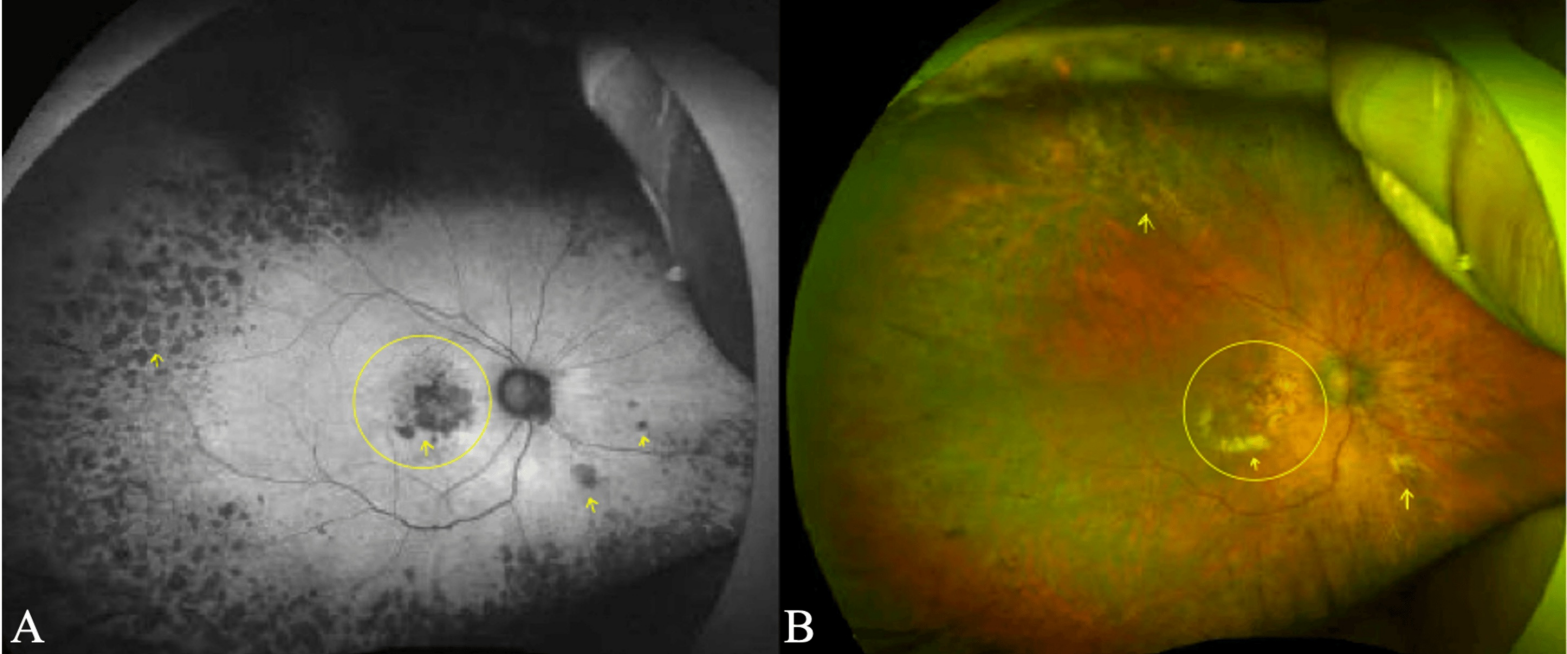
(A, B) OPTOS Ultra Wide Field (UWF) Image of the Right Eye (OD) Using Sensory Red-Free Filter The yellow arrow and circle indicate the presence of drusen distributed throughout the fundus.

**Figure 3:**
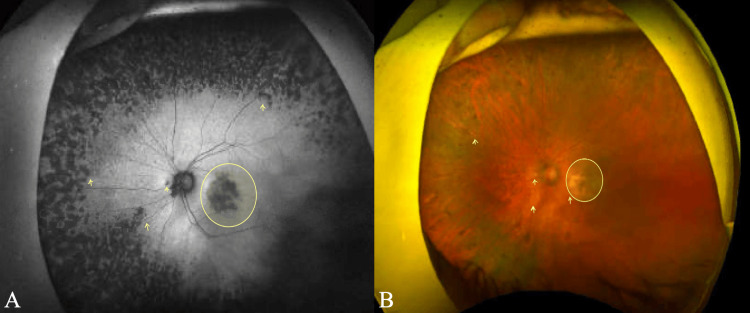
(A, B) OPTOS Ultra Wide Field (UWF) Image of the Left Eye (OS) Using a Sensory Red-Free Filter The yellow arrow and circle highlight the presence of drusen distributed throughout the fundus.

There was evidence of macular scarring with atrophy and the presence of choroidal neovascularization in the right eye (OD), accompanied by a small subretinal hemorrhage. In contrast, the left eye (OS) showed no evidence of subretinal fluid, hemorrhage, or neovascularization on OPTOS ultra-widefield (UWF) imaging. Nevertheless, the presence of widespread drusen indicates a potential risk for progression to the neovascular form. Fundus findings documented on the day of assessment are illustrated in Figures 2-3.

Optical coherence tomography (OCT) findings

OCT using the Carl Zeiss Meditec Cirrus HD-OCT 5000 was performed to evaluate the macula and vitreoretinal interface in both eyes. Imaging revealed a persistent posterior hyaloid membrane along with vitreomacular adhesion (VMA) bilaterally, indicating incomplete posterior vitreous detachment. These findings suggest early tractional changes that may contribute to visual distortion and reduced visual acuity.

Further analysis of the outer retina demonstrated signs of outer retinal tubulation (ORT), a degenerative feature commonly associated with advanced AMD, reflecting chronic photoreceptor disruption and remodeling. RPE atrophy was also observed, characterized by thinning and loss of the RPE layer, which is essential for photoreceptor support and function.

Additionally, multiple drusen were observed in both eyes (OU). These appeared as hyperreflective deposits located between the RPE and Bruch’s membrane. The coexistence of RPE atrophy, drusen, and outer retinal changes is indicative of a chronic degenerative process involving the central macula.

**Figure 4:**
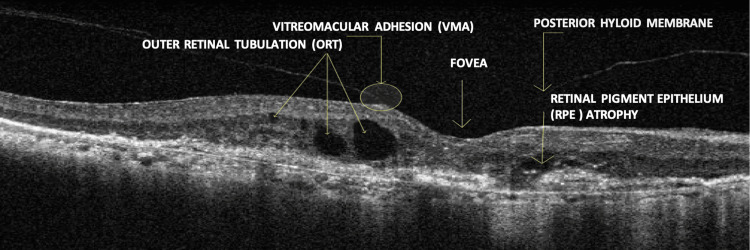
Carl Zeiss Meditec Cirrus HD-OCT 5000 Optical Coherence Tomography Image of the Right Eye (OD)

**Figure 5:**
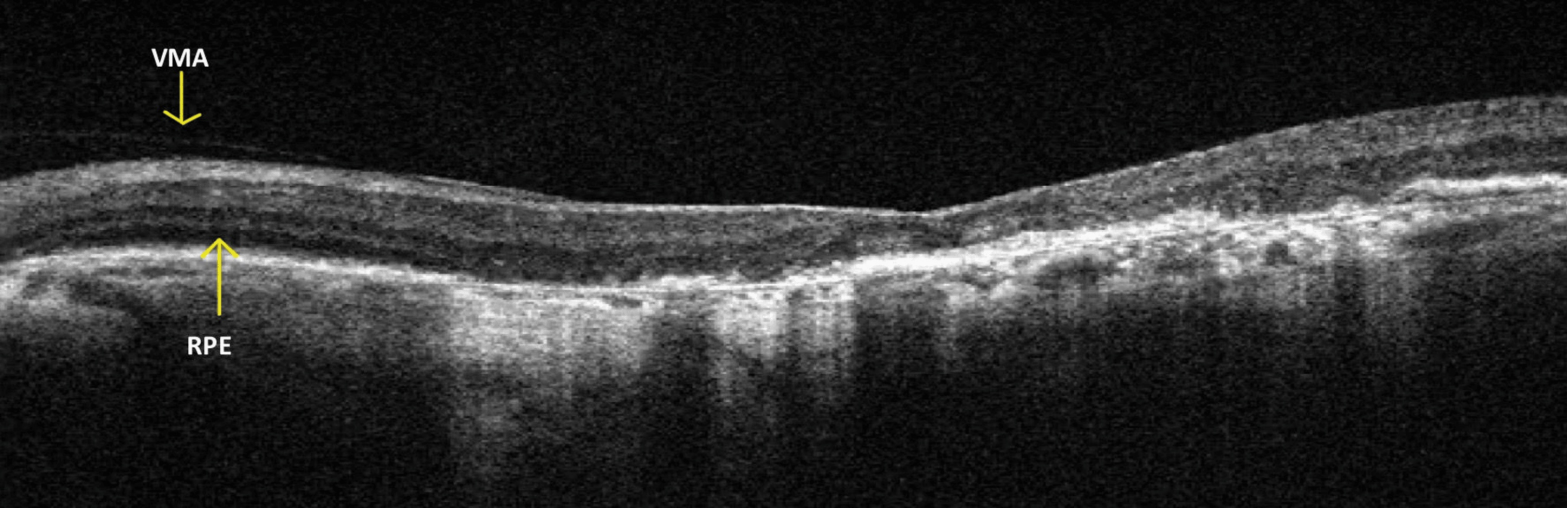
Carl Zeiss Meditec Cirrus HD-OCT 5000 Optical Coherence Tomography Image of the Left Eye (OS) VMA: vitreomacular adhesion; RPE: retinal pigment epithelium

Overall, the right eye (OD) demonstrated features consistent with neovascular (wet) AMD, while the left eye (OS) showed non-neovascular (dry) AMD without evidence of choroidal neovascular membrane (CNVM). These findings are illustrated in Figures 4-5.

Low vision assessment and intervention

During the low vision assessment, the patient presented with unaided distance visual acuity of 20/200 (6/60) in both eyes, indicating significant visual impairment. To address her distance vision needs, a 2.5× Galilean binocular telescope (Eschenbach 1639) was prescribed. This low vision aid provided effective magnification and enhanced visual clarity. With the telescope, her distance visual acuity improved to 20/40 (6/12) in both eyes. The patient reported a noticeable improvement in her ability to see clearly and described the viewing experience as significantly more comfortable and satisfactory.

For near vision, unaided near visual acuity was measured at 3.2M (approximately 20/160, equivalent to 6/48) in both eyes, consistent with difficulty performing reading and other close tasks. To assist with near activities, a 5× (20-diopter) illuminated hand magnifier (Eschenbach Mobilux 15105) was prescribed and used during the evaluation. With this aid, her near visual acuity improved to 1.0M in both eyes (approximately 20/50, equivalent to 6/18+), allowing her to perform near tasks more effectively and with greater ease. 

The differential diagnosis included uncorrected refractive error, macular degeneration, diabetic retinopathy, and cataracts, all of which can cause persistent blurring of vision at both distance and near. Cataracts, often age-related, result from lens opacification and are known to reduce visual clarity across all focal ranges; however, in this patient, the ocular media were clear on examination, and visual impairment did not improve with refractive correction alone, suggesting an underlying retinal pathology.

Central serous chorioretinopathy (CSR) was also considered, as it can share overlapping features with neovascular AMD, such as subretinal fluid and visual disturbances. CSR typically affects younger males and is characterized by localized serous detachment of the neurosensory retina, often in association with stress or corticosteroid use [3]. On OCT, CSR demonstrates a dome-shaped retinal elevation with subretinal fluid and minimal intraretinal changes, features not observed in this patient. Instead, OCT revealed subretinal hyperreflective material and pigment epithelial detachment, findings more consistent with choroidal neovascularization characteristic of wet AMD [3]. While CSR was ruled out as the primary diagnosis, subtle irregularities of the RPE supported its inclusion as an extended differential diagnosis.

Stargardt’s disease, the most common inherited macular dystrophy, was also considered. Typically presenting in childhood, it is characterized by foveal atrophy and scattered, pale yellow, fleck-like deposits in the retina [4]. Although the patient reported blurred vision at both distance and near, the age of onset and absence of classic retinal findings made Stargardt’s disease less likely.

Management and treatment plan

The patient was counseled that the primary cause of her visual impairment is AMD, with the right eye affected by wet AMD and the left eye by dry AMD, as confirmed by clinical examination. The impact of the disease on central vision, including the potential development of a central scotoma, was clearly explained.

Low vision optical aids were prescribed for both distance and near tasks. These devices were demonstrated in-office with detailed instructions on their intended use to compensate for central vision loss and enhance functional vision. Visual acuity testing confirmed the benefit of these aids.

The patient was referred to low vision rehabilitation services and ophthalmology for ongoing care and training in adaptive strategies to maximize her remaining vision. Routine follow-up was advised to monitor disease progression and to detect any early signs of conversion to neovascular (wet) AMD in the left eye under ophthalmology management.

She was subsequently referred to a retina specialist at a tertiary ophthalmology clinic for further management of neovascular AMD in the right eye. Treatment was initiated with aflibercept (Eylea), administered as an inferotemporal intravitreal injection of 2 mg (0.05 mL). A loading regimen consisting of three consecutive monthly intravitreal injections was given to control choroidal neovascular activity and reduce the risk of further visual deterioration.

Environmental modifications were discussed, including optimizing lighting conditions for near activities and reducing glare to improve visual comfort. Additional low vision aids were recommended, including high-contrast and large-print reading materials, yellow filters for indoor contrast enhancement, plum filters for outdoor glare control and contrast sensitivity, and closed-circuit television (CCTV) systems to provide magnification and improve near-task performance.

Educational counseling was provided to ensure the patient’s understanding of her condition and management plan. Support resources were offered to assist with psychological adaptation and to connect the patient with relevant services.

All prescribed devices and interventions were thoroughly reviewed to promote informed engagement and adherence.

First follow-up visit

The patient returned to the clinic for a follow-up examination on February 6, 2024, approximately three months after the initial low vision rehabilitation assessment. Visual acuity remained stable, with no significant changes or disease progression noted after the injection. OCT and fundus photography demonstrated consistent retinal findings compared to the prior evaluation, with no evidence of worsening.

The patient reported effective management of daily activities using the prescribed low vision aids and expressed satisfaction with the current rehabilitation regimen. The existing low vision management plan continues to be appropriate for maintaining functional vision and supporting independence.

Ongoing monitoring is recommended to detect any future changes in visual function or signs of progression, especially conversion to neovascular (wet) AMD in the left eye. The patient was advised to adhere to regular follow-up visits and to continue utilizing the prescribed visual aids and adaptive strategies.

## Discussion

AMD is a leading cause of irreversible central vision loss in the elderly population [5]. The dry (non-neovascular) form accounts for the majority of cases and is characterized by the accumulation of drusen, geographic atrophy of the RPE, and photoreceptor degeneration in the macula [2,6]. These degenerative changes lead to progressive central vision loss, significantly impairing a patient’s ability to read, recognize faces, and perform tasks requiring fine visual discrimination.

In this case, a 70-year-old female presented with reduced visual acuity at both distance and near, measuring 20/200 (6/60) OU unaided. Near visual acuity was 3.2M (20/160, 6/48). With appropriate refractive correction, best-corrected visual acuity improved to 20/125 (6/37.5) OU, indicating a coexisting refractive component in addition to macular pathology. Amsler grid testing revealed central distortion and scotomas OU, consistent with macular dysfunction secondary to AMD.

Further evaluation using OCT and widefield fundus imaging revealed bilateral drusen, RPE atrophy, and VMA, findings consistent with AMD [6,7]. Contrast sensitivity was also reduced (20/100, 6/30), indicating significant functional visual impairment and highlighting the need for visual rehabilitation. Overall, the right eye (OD) exhibited features suggestive of neovascular (wet) AMD, with evidence of a CNVM, whereas the left eye (OS) demonstrated non-neovascular (dry) AMD without evidence of CNVM.

Management focused on a multi-modal low vision rehabilitation plan designed to enhance functional vision and independence. Prescribed optical devices included the Eschenbach 1639 2.5× Tele-Comfort Galilean binocular telescope for distance (improving acuity to 20/40, 6/12) and the Mobilux illuminated 5× (20 D) hand magnifier for near, which enabled reading 1.0M print (approximately 20/50, equivalent to 6/18+). A near addition of +6.25 D was used for smaller print, while +3.00 D was sufficient for 2.5M text. Additional aids included contrast-enhancing filters (yellow for indoor use, plum for outdoor use), task lighting, glare-reduction strategies, and the use of CCTV systems for variable magnification.

Visual field testing in AMD typically reveals central scotomas and reduced macular sensitivity due to photoreceptor loss and RPE dysfunction. In some cases - particularly in advanced or neovascular forms - peripheral field involvement may also occur. This patient exhibited scotomas and reduced central sensitivity consistent with AMD in both eyes (OU), which is characterized by the presence of medium-to-large drusen and/or RPE pigmentary abnormalities without evidence of advanced geographic atrophy or choroidal neovascularization [6]. For this patient’s right eye (OD) with neovascular (wet) AMD, intravitreal aflibercept (Eylea) was an appropriate therapeutic choice. The literature provides strong evidence that anti-VEGF agents, including aflibercept (Eylea), are effective in controlling choroidal neovascularization and improving or stabilizing visual acuity. Following the standard loading regimen of three consecutive monthly injections, numerous studies have revealed significant anatomical improvement and favorable visual outcomes. In this case, the treatment resulted in stabilization of the patient’s visual acuity, consistent with outcomes reported in the literature [8].

Fundus imaging revealed bilateral optic nerve heads with a C/D ratio of 0.4 and well-defined margins. Although the NRR follows the ISNT rule, intraocular pressure and retinal nerve fiber layer (RNFL) thickness were within normal limits, suggesting no current glaucomatous damage [9-12]. Nonetheless, this anatomical deviation warrants continued surveillance. Widespread drusen were noted across both the macula and peripheral retina in all gaze positions, consistent with AMD and indicating an elevated risk for progression. Additionally, a tigroid fundus appearance, likely due to RPE thinning and choroidal visibility, further supported chronic degenerative changes.

Importantly, the presence of VMA observed on OCT raises concern for potential progression to vitreomacular traction (VMT) and its associated sequelae. VMA refers to an incomplete separation of the posterior vitreous cortex from the macula, where persistent adherence can exert anterior-posterior tractional forces. If this traction increases, it may lead to foveal distortion, worsening central vision, or even the development of a full-thickness macular hole [6,9]. Such complications can result in significant visual impairment and may necessitate surgical intervention, such as pars plana vitrectomy.

In addition, ORTs, tubular structures observed in chronic retinal degeneration, were identified on OCT. These findings are indicative of photoreceptor layer remodeling and are most commonly associated with advanced stages of dry AMD. While the overall clinical presentation remains consistent with wet AMD in the right eye (OD) and dry AMD in the left eye (OS), the presence of ORT suggests focal areas of localized photoreceptor degeneration. Taken together, the presence of VMA and ORT highlights the importance of serial OCT monitoring to track structural changes and detect tractional complications at an early stage [6].

Comprehensive management of dry and wet AMD includes regular monitoring using tools such as the Amsler grid, periodic refraction to optimize visual acuity, and referral to low vision rehabilitation services for training in adaptive techniques [10]. Patient education is essential to promote effective self-monitoring and timely reporting of any vision changes, particularly for early detection of conversion to wet AMD, such as in the right eye (OD), or complications such as VMT or macular hole formation.

Emerging therapies, including photobiomodulation and gene therapies targeting the complement pathway, are under investigation and may offer additional treatment options in the future [11]. Nutritional supplementation with the AREDS (Age-Related Eye Disease Study) formulation has demonstrated efficacy in slowing progression in intermediate AMD [6], and the well-established association between smoking and AMD reinforces the importance of smoking cessation [6]. This patient denies a history of smoking and may benefit from micronutrient supplementation.

By integrating optical low vision devices, environmental modifications, and ongoing clinical follow-up, the patient has achieved meaningful improvements in visual function [10], supporting greater independence and quality of life despite central vision loss.

## Conclusions

This case demonstrates the role of low vision rehabilitation in a patient with neovascular (wet) AMD in the right eye and non-neovascular (dry) AMD in the left eye. The use of optical aids, including a Galilean telescope for distance vision and an illuminated hand magnifier for near tasks, resulted in improved device-assisted visual acuity and reading performance. These findings highlight the potential benefit of individualized low vision aids in improving measurable visual function in patients with AMD. Ongoing follow-up is important for monitoring disease progression and managing potential complications.
